# The Outcome of 270-Degree Selective Laser Trabeculoplasty

**DOI:** 10.1155/2012/313616

**Published:** 2012-12-05

**Authors:** Pasi Tapio Pehkonen, Juha Oskari Välimäki

**Affiliations:** Department of Ophthalmology, Päijät-Häme Central Hospital, Keskussairaalankatu 7, 15850 Lahti, Finland

## Abstract

*Purpose*. To evaluate the reduction of intraocular pressure (IOP) by a single-session 270° selective laser trabeculoplasty (SLT) in pseudoexfoliation glaucoma (PXFG) and primary open angle glaucoma (POAG) patients. *Methods*. A successful outcome was defined as an IOP reduction ≥20% from baseline with no further need for laser or incisional surgery. The preoperative pharmaceuticals were maintained unchanged throughout the course of the study. 70–80 nonoverlapping pulses were distributed around 270° in the trabecular band. *Results*. Sixty-six eyes of 42 patients with PXFG (30 eyes) or POAG (36 eyes) met the inclusion criteria. The mean ± standard deviation preoperative IOP was 23.7 ± 4.5 mmHg and at the end of the followup was 19.0 ± 4.5 mmHg with a pressure drop of 4.7 ± 3.1 mmHg (20%) (*P* < 0.001, 95% confidence interval 3.94–5.46). The cumulative probability of success was 39% (26 of 66 eyes) after 6 months of followup. Statistically significant differences in success rates were observed between the PXFG and POAG groups (27% versus 50%; *P* = 0.025). Postoperative inflammatory reaction was scanty. *Conclusions*. 270-degree SLT is useful in lowering IOP in PXFG and POAG, but the average reduction of IOP seems to be within the same range as reported with 180-degree SLT previously.

## 1. Introduction

Selective laser trabeculoplasty (SLT) uses *Q*-switched, 532 nm Nd:YAG laser pulses that are selectively absorbed by melanin-containing cells without damaging nonmelanin containing cells in the trabecular meshwork [1]. At present, it is not known which is the best extent of a single-session SLT treatment in the trabecular band. Theoretically, it would be safer to treat less than 360-degree during a single SLT session.

Nagar and coworkers reported that SLT treatment of 90° was significantly less effective than treatment of 180° or 360° in patients with open angle glaucoma [2]. According to the same study, no statistically significant difference in success rates was found between 180° and 360° SLT treatments. However, there are also reports in which SLT covering 360 degrees of the trabecular meshwork is stated to be more effective than 180-degree treatment as a primary therapy or an adjunctive treatment [3, 4]. 

To the best of our knowledge, there are no published prospective, clinical studies considering the 270-degree SLT treatment. The purpose of this prospective study was to analyze the pressure lowering potential of a 270-degree single-session SLT treatment in pseudoexfoliation glaucoma (PXFG) and primary open angle glaucoma (POAG) patients with unchanged glaucoma medication during a 6-month followup. 

## 2. Materials and Methods

This single-centre, prospective, nonrandomized comparative study was performed between January 2009 and March 2010 at the Päijät-Häme Central Hospital. The Ethics Committee of the Päijät-Häme Central Hospital approved the study protocol. All patients were interviewed and informed and gave their verbal and written informed consent before enrollment. Participation in the study was voluntary. 

Eligibility criteria included a diagnosis of PXFG or POAG requiring therapy in addition to their current antiglaucoma medications and patients being over 18 years of age. Patients were excluded if the trabecular meshwork could not be viewed at 360 degrees, if they had used systemic or topical steroids, had any surgery or ALT of the eye within the previous six months, or if they had ocular trauma or any other preexisting corneal disease precluding the angle evaluation and treatment.

Forty-two consecutive patients (66 eyes) fulfilling the inclusion criteria were entered into the study. Patients received first-time SLT procedure if their glaucoma was uncontrolled with maximum tolerated medical management. One surgeon (PP) performed all laser treatments. All study eyes underwent their SLT with the *Q*-switched frequency-doubled 532 nm Nd : YAG laser (Ellex Tango SLT/Photodistruptor Combination Laser, Ellex Medical Pty Ltd, Australia) with pulse duration of 3 ns and a spot size of 400 microns. 

No additional pretreatment with antiglaucoma medication was used. After topical anaesthesia oxybuprocaine hydrochloride 4 mg/mL, goniolens (Ocular Latina SLT Gonio Laser Lens, Ocular Instruments, USA) with methylcellulose was used to focus a laser aiming beam to the trabecular meshwork. A train of 70–80 nonoverlapping pulses was evenly distributed around 270° in the trabecular band. The laser's energy was initially set at 0.3 mJ per pulse and increased by 0.1-0.2 mJ until small “champagne bubbles” were observed and then decreased by 0.1 mJ. All patients were given ketorolac tromethamine 0.5% (Acular, Allergan Pharmaceuticals Ireland, Ireland) drops 4 times daily for 7 days postoperatively and instructed to continue routine glaucoma medications.

Patients were postoperative examined after 1 day, 1 week, and 1, 3, and 6 months. At each postoperative visit, IOP and anterior chamber inflammation reaction were examined and data from the glaucoma medication and further surgical procedures were collected. Also intra- and postoperative complications were observed. In this study, all IOP measurements were done using the same Goldman applanation tonometer.

The eye was regarded as being successfully treated with SLT when the following conditions were met: an IOP reduction ≥20% from baseline without changing the antiglaucoma medication, no need for further laser trabeculoplasty or glaucoma surgery. The preoperative pharmaceuticals were maintained unchanged throughout the course of the study. The protocol included a safety hatch that entailed an exception to the principle of unchanged glaucoma medication. If the posttreatment IOP at 1- or 3-month visit exceeded the preoperative IOP value, additional glaucoma surgery was done. Preoperative IOP and the total number of glaucoma medication were calculated on the day of SLT treatment.

Statistical analyses were performed using SPSS version 13.0 for Windows (SPSS Inc., Chicago, IL, USA). Continuous variables were analyzed using two-tailed *t*-tests and categorical variables with the Mann-Whitney *U* test. The Kaplan-Meier life-table analysis was used to determine the surgical outcome. The authors followed the Tenets of the Declaration of Helsinki.

## 3. Results

SLT was performed on 66 eyes of 42 patients with PXFG (Group A = 30 eyes) or POAG (Group B = 36 eyes). The treatment groups did not differ in baseline characteristics, except there was significant difference between the mean preoperative IOP (*P* = 0.005) (Table 1). During the followup time, no additional surgery or laser treatment was performed on any of the eyes of the study groups. The mean energy used in the PXFG group was 39.7 ± 9.5 mJ (range 28–65 mJ) and in the POAG group 45 ± 10.6 mJ (range 25–74 mJ).

The mean IOP ± standard deviation (SD) preoperatively in 66 eyes was 23.7 ± 4.5 mmHg (range 14–37 mmHg), and at the end of the followup 19.0 ± 4.5 mmHg (range 10–32 mmHg), with a pressure drop of 4.7 ± 3.1 mmHg (20%). The difference in IOP was statistically significant (*P* < 0.001, 95% confidence interval 3.94–5.46, paired *t*-test). An elevation in IOP was not detected on day 1 in any of the study eyes. On day 7, an elevation in IOP was noted in 4 eyes in the PXFG group, the maximum elevation being 2 mmHg. 

The IOP reductions from preoperative to postoperative at 1 day, 1 week, 1 month, 3 months, and 6 months were statistically significantly different both in Group A and Group B (Tables 2 and 3).  Table 4 shows IOP variation in Group A and Group B at baseline and at various times after the treatment. Only 17 of 66 eyes (26%) had ≥3 antiglaucoma medications at the baseline. We compared the eyes on ≥3 antiglaucoma medications preoperatively to the eyes on a lower number of medications and they had the mean IOP of 18.3 ± 5.3 mmHg and 19.3 ± 4.1 mmHg, respectively, at 6-month followup. No significant difference was demonstrated in the IOP at the end of the followup between these two groups (*P* = 0.501, independent samples *t*-test). 

Of all eyes undergoing SLT, 27 (41%) had a preoperative IOP ≥25 mmHg. Fourteen of those 27 eyes (52%) and 12 of 39 eyes (31%) with IOP <25 mmHg were classified as success. Eyes with a higher preoperative IOP had a statistically significantly better success rate than eyes with a preoperative IOP <25 mmHg (*P* = 0.042, log-rank test). None of the study patients needed extra surgery during the course of the study.

**Figure 1:**
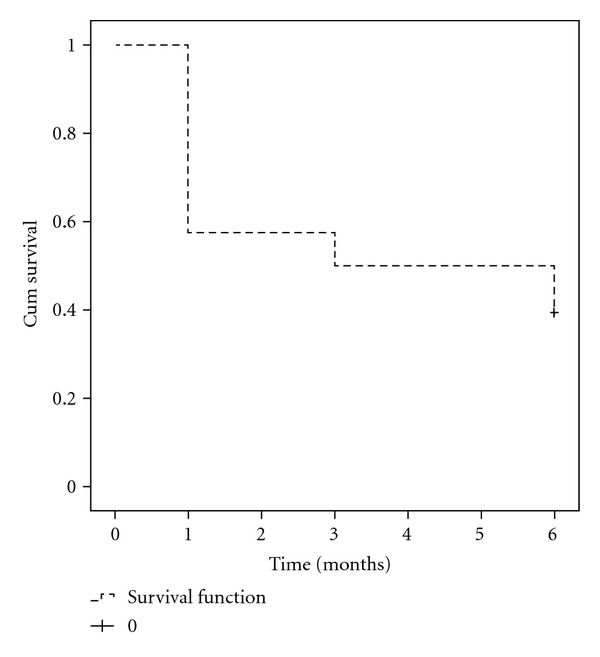
Kaplan-Meier survival curve for 66 eyes of 42 patients after 270° SLT treatment. Success was defined as an IOP reduction ≥20% from baseline without changing the antiglaucoma medication with no need for further laser trabeculoplasty or glaucoma surgery.

**Figure 2:**
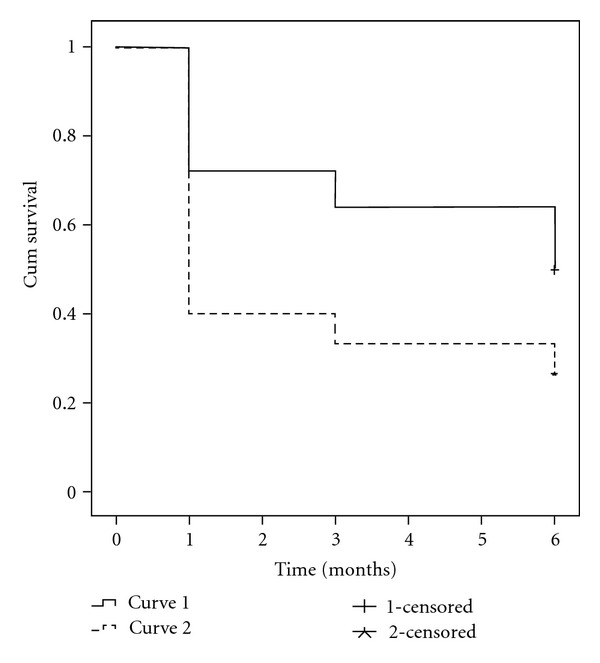
Kaplan-Meier survival curves for eyes with POAG (Curve 1) versus eyes with PXFG (Curve 2) with the success criteria defined as an IOP reduction ≥20% from baseline without changing the antiglaucoma medication with no need for further laser trabeculoplasty or glaucoma surgery; *P* = 0.025 log rank test.

The overall success rate was 39% (26 of 66 eyes) after 6 months of followup. Only 19 of 66 eyes (29%) maintain the postlaser IOPs ≤18 mmHg. A Kaplan-Meier life-table analysis of the study patients is presented in Figure 1. The cumulative probability of success was 27% in Group A and 50% in Group B (Figure 2). Statistically significant differences in success rates between Group A and Group B were observed using the log-rank test (*P* = 0.025). There were no intra- or postoperative complications due to SLT. None of the study eyes had flare/cells either at the beginning or at the first-month followup visit. 

## 4. Discussion

This prospective study demonstrated a mean IOP reduction of 4.7 mmHg (20%) after 270-degree SLT treatment in patients with PXFG and POAG. The average reduction of IOP after 180-degree SLT has been reported to vary between 4.6 mmHg and 5.06 mmHg (18.7%–22.5%) after 6 months of followup, which is within the same range as in the present study with 270-degree SLT [5–7]. Compared to those studies, more patients with PXFG were present in our study. As a primary therapy, the mean reduction in IOP has been reported to be as high as 8.9 mmHg (35%) after 360-degree SLT in patients with POAG and ocular hypertension [3]. 

Although it is only a speculation, the quite similar IOP reduction after 270-degree SLT than after 180-degree SLT could be explained by higher number of advanced glaucoma in our study. Patients with advanced glaucoma tend to get a high failure rate. Unfortunately, the severity of the glaucoma was not evaluated in this study. Another explanation to the similar results might be that there was lower preoperative IOP in the present study compared to the study by Latina et al. [5] with 180-degree SLT.

According to the retrospective study of Výborný and Sicáková [8] with 270-degree SLT in POAG patients, the mean decrease of the IOP was lower than in our study (3.3 mmHg versus 5.1 mmHg, resp.) after 6 months followup [3]. We did not use fixed laser power (1 mJ) in every study patient as Výborný and Sicáková did in their study. In addition, only in the present study the glaucoma medication was unchanged during the followup.

With 270-degree treatment, the postoperative IOP elevation was not clinically significant on day 1 or day 7. There might have been some study eyes with a spike in IOP during the first postoperative 24 hours but the elevation was short-lived and passed away before the measurement on day 1. Nagar and coworkers reported an IOP elevation > 5 mmHg at 1-hour postoperative in 8 eyes (16%) after 180° SLT and in 12 eyes (27%) after 360° treatment [2]. 

The Advanced Glaucoma Intervention Study showed that in patients in whom IOP was constantly kept less than 18 mmHg, progression of visual field loss was minimal [9]. Although no particular level of IOP can be considered universally safe for all glaucoma patients, only 29% of the study eyes maintain the postlaser IOPs less than or equal to 18 mmHg. The grade of glaucoma was not determined in study patients; however, none of the study patients had early manifest of open angle glaucoma. According to our results, 270-degree SLT seems not to be effective as an adjunctive therapy to the antiglaucoma medication to achieve the target pressure lower than 19 mmHg. 

In our study, eyes with a higher preoperative IOP (≥25 mmHg) seem to have a tendency for a better success rate after 270° SLT procedures. Thomas and coworkers reported in patients treated with ALT that the amount of IOP reduction is directly related to the height of the prelaser IOP: the higher the baseline IOP, the greater the postlaser IOP drop [10]. The results of the present study confirms that conclusion. However, according to our study, the mean pressure reduction was 20%, which may not be enough in patients with IOP over 30 mmHg. Therefore, 270-degree SLT might be most beneficial in eyes with IOP ranging from 25 mmHg to 30 mmHg. 

According to our results, eyes with POAG seem to have better life-table success rate than eyes with PXFG after 270-degree SLT (50% versus 27%, resp.). However, the absolute IOP reduction from baseline to the last followup visit is around the same 5 mmHg in both PXFG and POAG groups at the present study. This is in agreement with previous report with SLT treatment performed 180° in PXFG and POAG eyes [11]. Our study implies that eyes with PXFG fail sooner than eyes with POAG after SLT treatment, which is similar to what is reported with ALT. The initial IOP reduction to ALT has been reported to be greater in PXFG than POAG eyes, but after long-term followup period, there was no significant difference [12]. 

We did not find any intra- or postoperative SLT complications. Inflammatory reactions, cells, and flare, with SLT, were scanty indicating minimal thermal effect on the trabecular meshwork. The fixed-medication feature during the followup offered us the opportunity to evaluate the success of IOP reduction due to absorption of the laser energy. The major weaknesses of our study are the small numbers of study patients, a relatively short followup of six months only, and the fact that the severity of glaucoma was not evaluated. However, from the ethical standpoint, it might be a problem to keep glaucoma medication unchanged longer than a 6-month followup period in case there is no IOP reduction response to SLT treatment. 

ALT has been assumed to cause more structural damage to the eyes in comparison to newer laser techniques, such as SLT or micropulse diode laser trabeculoplasty (MDLT) [13, 14]. Patients with maximal or near-maximally tolerated antiglaucoma medication do not appear to respond as well to LTP as patients with lower numbers of antiglaucoma medication preoperatively [15]. In the present study, eyes with a lower number of medications did not have a better success rate than eyes with ≥ 3 medications. This observation was in agreement with our previous study on 180-degree MDLT treatment [16]. 

In conclusion, the results of this prospective clinical study suggest that 270-degree SLT is an effective and safe supplemental treatment for patients with POAG and PXFG. However, the average reduction of IOP after 270-degree SLT is within the same range as reported with 180-degree SLT previously. 

## Figures and Tables

**Table 1:** Demographic data of the study population.

	Group A *n* = 30	Group B *n* = 36	*P* value
IOP (mean ± SD), mmHg	22.0 ± 4.0	25.1 ± 4.6	0.005
Age (mean ± SD), y	71 ± 10	68 ± 10	0.216
Sex (men) (%)	7 (23%)	14 (39%)	0.178
Eye (right) (%)	15 (50%)	18 (50%)	0.884
No. of medication (mean ± SD)	1.63 ± 1.22	1.72 ± 1.32	0.777
No. of previous argon laser trabeculoplasty (mean ± SD)	0.23 ± 0.43	0.11 ± 0.32	0.203
No. of previous operations (mean ± SD)	0.23 ± 0.43	0.19 ± 0.40	0.720
No. of glaucoma operations (mean ± SD)	0.10 ± 0.30	0.03 ± 0.17	0.252
Pseudophakic* (%)	7 (23%)	5 (14%)	0.310

Group A: pseudoexfoliation glaucoma; Group B: primary open angle glaucoma; SD: standard deviation; IOP: intraocular pressure.

*None of study patients were aphakic.

**Table 2:** IOP reduction from baseline at each time interval in pseudoexfoliation glaucoma group (Group A).

Time interval	*n*	Mean IOP reduction ± SD (%) mmHg	*P* value
Pre-SLT to day 1	30	4.9 ± 2.6 (22.2)	<0.001
Pre-SLT to 1 week	30	2.3 ± 2.7 (10.4)	<0.001
Pre-SLT to 1 month	30	3.5 ± 2.9 (15.9)	<0.001
Pre-SLT to 3 months	30	4.6 ± 2.6 (20.9)	<0.001
Pre-SLT to 6 months	30	4.2 ± 3.2 (19.1)	<0.001

IOP: intraocular pressure; SD: standard deviation; SLT: selective laser trabeculoplasty.

**Table 3:** IOP reduction from baseline at each time interval in primary open angle group (Group B).

Time interval	*n*	Mean IOP reduction ± SD (%) mmHg	*P* value
Pre-SLT to day 1	36	8.4 ± 4.9 (33.5)	<0.001
Pre-SLT to 1 week	36	5.2 ± 3.9 (20.7)	<0.001
Pre-SLT to 1 month	36	6.1 ± 3.6 (24.3)	<0.001
Pre-SLT to 3 months	36	6.5 ± 3.1 (25.9)	<0.001
Pre-SLT to 6 months	36	5.1 ± 3.0 (20.3)	<0.001

IOP: intraocular pressure; SD: standard deviation; SLT: selective laser trabeculoplasty.

**Table 4:** IOP variation in group A and group B at baseline and at various times after the SLT treatment.

Time	Group A (*n* = 30) Mean IOP ± SD (range) mmHg	Group B (n = 36) Mean IOP ± SD (range) mmHg
Baseline	22.0 ± 4.0 (14–32)	25.1 ± 4.6 (17–37)
Day 1	17.1 ± 3.4 (10–23)	16.7 ± 4.5 (7–32)
1 week	19.7 ± 4.0 (12–31)	20.0 ± 4.1 (12–31)
1 month	18.5 ± 3.3 (12–26)	19.0 ± 3.7 (12–30)
3 months	17.4 ± 3.2 (12–24)	18.6 ± 3.6 (12–25)
6 months	17.8 ± 3.6 (10–27)	20.0 ± 4.9 (13–32)

IOP: intraocular pressure; Group A: pseudoexfoliation glaucoma; Group B: primary open angle glaucoma; SD: standard deviation.
